# Transmembrane Helices 2 and 3 Determine the Localization of Plasma Membrane Intrinsic Proteins in Eukaryotic Cells

**DOI:** 10.3389/fpls.2019.01671

**Published:** 2020-01-10

**Authors:** Hao Wang, Liyuan Zhang, Yuan Tao, Zuodong Wang, Dan Shen, Hansong Dong

**Affiliations:** ^1^ Department of Plant Pathology, Nanjing Agricultural University, Nanjing, China; ^2^ Department of Plant Pathology, Shandong Agricultural University, Taian, China; ^3^ State Key Laboratory of Crop Biology, Taian, China

**Keywords:** aquaporin, AtPIP1, AtPIP2, localization, transmembrane helices

## Abstract

In plants, plasma membrane intrinsic protein (PIP) PIP1s and PIP2s mediate the transport of disparate substrates across plasma membranes (PMs), with a prerequisite that the proteins correctly localize to the PMs. While PIP2s can take correct localization by themselves in plant cells, PIP1s cannot unless aided by a specific PIP2. Here, we analyzed the localization of the *Arabidopsis* aquaporins, AtPIP1s, AtPIP2;4, and their mutants in yeast, *Xenopus* oocytes, and protoplasts of *Arabidopsis*. Most of AtPIP2;4 localized in the PM when expressed alone, whereas AtPIP1;1 failed to realize it in yeast and *Xenopus* oocytes. Switch of the transmembrane helix 2 (TM2) or TM3 from AtPIP1;1 to AtPIP2;4 disabled the latter’s PM targeting activity. Surprisingly, a replacement of TM2 and TM3 of AtPIP1;1 with those of AtPIP2;4 created a PM-localized AtPIP1;1 mutant, 1;1Δ(TM2+TM3)/2;4(TM2+TM3), which could act as a water and hydrogen peroxide channel just like AtPIP2;4. A localization and function analysis on mutants of AtPIP1;2, AtPIP1;3, AtPIP1;4, and AtPIP1;5, with the same replaced TM2 and TM3 from AtPIP2;4, showed that these AtPIP1 variants could also localize in the PM spontaneously, thus playing an inherent role in transporting solutes. Sequential and structural analysis suggested that a hydrophilic residue and a defective LxxxA motif are modulators of PM localization of AtPIP1s. These results indicate that TM2 and TM3 are necessary and, more importantly, sufficient in AtPIP2 for its PM localization.

## Introduction

Aquaporins (AQPs) are transmembrane channel proteins with a broad variety of biological functions in all kingdoms. AQPs were found to facilitate water diffusion across cell membranes when expressed in *Xenopus laevis* oocytes (Agre et al., 1993). In recent years, some other uncharged small solutes, such as hydrogen peroxide (H_2_O_2_), glycerol, urea, and ammonia, are also proved to be the substrates of AQPs (Soria et al., 2010; Benga, 2012; Kirscht et al., 2016; Tian et al., 2016). Furthermore, AQPs play important roles in resistance regulation, signal transduction, nutrient uptake, and transduction in various organisms (Chaumont et al., 2017).

Plant AQPs include plasma membrane intrinsic proteins, nodulin26-like intrinsic proteins (NIPs), the small basic intrinsic proteins (SIPs), the tonoplast intrinsic proteins (TIPs), and the poorly characterized X intrinsic proteins (XIPs). (Chaumont et al., 2001; Johanson et al., 2001; Danielson and Johanson, 2008). PIPs are divided into two major groups, PIP1 and PIP2, which share about 80% amino acid identity. Despite such a high similarity, PIP1s and PIP2s show different transmembrane water fluxes when expressed in *X. laevis* oocytes (Kammerloher et al., 1994; Chaumont et al., 2000; Yaneff et al., 2015). In correspondence with this, PIPs show a difference in subcellular localization between PIP1 and PIP2 groups, which explains the difference of water transporting activity in *X. laevis* oocytes (Fetter et al., 2004; Yaneff et al., 2015; Bienert et al., 2018). PIP2s are able to massively traffic to the plasma membrane of *X. laevis* oocytes and serve as functional transmembrane channels, while PIP1s showed little localization in the PM and a significant lower transporting capacity if expressed alone (Kammerloher et al., 1994; Fetter et al., 2004).

Generally, the newly synthesized and properly folded PM proteins will be transported from endoplasmic reticulum (ER) to Golgi and finally to PM *via* the vesicle system (Demmel et al., 2011; Liu and Li, 2014; Geva and Schuldiner, 2014). However, the PIP1s are going to be detained in intracellular membranes, especially endoplasmic reticulum membranes (Zelazny et al., 2007; Chevalier et al., 2014). In PIP1s, the missing PM trafficking signal in transmembrane helix 3 makes it hard to be successfully secreted from ER (Chevalier and Chaumont, 2015). Interestingly, when expressed together with PIP2s in oocytes, PIP1s successfully localized in the PM just like PIP2s (Fetter et al., 2004). PIP1s interact with PIP2s and form stable heterotetramers. The heterotetramerization between PIP1s and PIP2s enables PIP1s to be secreted along with PIP2s from ER to PM (Zelazny et al., 2007; Chevalier et al., 2014; Bienert et al., 2018). The massive PM localization brings PIP1s with the normal function of transporting water like PIP2s (Berny et al., 2016; Bienert et al., 2018). Therefore, PIP1 group owes an inherent capacity to transport substrates. But trafficking to the correct position is the prerequisite before PIP1 is able to carry out its full function.

Diacidic DxE (D, aspartic acid; E, glutamic acid; x, uncertain amino acid residue), buried within the N-terminal region, is characterized as a PM trafficking motif in plant PIPs (Zelazny et al., 2009; Sorieul et al., 2011). Wild-type ZmPIP2;4 and ZmPIP2;5 targeted to PM but are retained in ER if the DxE motif is mutated. However, the replacement of the N-terminal region of ZmPIP1;2 with that of ZmPIP2;5 which contains a DxE motif is not enough to enable ZmPIP1;2 in targeting to PM like ZmPIP2;5 (Zelazny et al., 2009). LxxxA (L, leucine; x, undetermined amino acid residue; A, alanine) in TM3 of ZmPIP2;5 is also essential in ER-to-Golgi trafficking, and considered to be an ER export signal (Chevalier et al., 2014). LxxxA motif mutant is not secreted from the ER. Nevertheless, replacement of both N-terminal and TM3 region of ZmPIP1;2 with those of ZmPIP2;5 did not yield successful PM localization (Chevalier et al., 2014). Some phosphorylation sites, such as S280 and S283 in the C-terminus of *Arabidopsis* PIP2;1, are also identified as candidates that can affect PIPs trafficking (Prak et al., 2008). In addition, some E3 ubiquitin ligases localized in the ER membrane could play a role in AtPIP2;1 trafficking by posttranslational modification (Lee et al., 2009). Moreover, the PM-localized SNARE isoform SYP121 regulates the transfer of PIPs from the vesicles to the PM (Besserer et al., 2012; Hachez et al., 2014).

However, although several motifs are characterized as affecting the localization of PIP2 group, what really determines the defect of PIP1 group is not fully understood. Here, we took AtPIP1;1 (AT3G61430) and AtPIP2;4 (AT5G60660) from *Arabidopsis thaliana* ecotype Col-0 on behalf of PIP1 isoforms and PIP2 isoforms respectively to investigate the contributing factors of their localization differences. In *A. thaliana*, there are 13 AtPIPs isoforms, 5 AtPIP1 isoforms, and 8 AtPIP2 isoforms, which are typical AQPs with six TMs and five loops. AtPIP2;4 is considered to be a water channel like other AtPIP2s and was also found to play a role in hydrogen peroxide transporting and response to abscisic acid (Kammerloher et al., 1994; Lewis et al., 1997; Dynowski et al., 2008; Kline et al., 2016). As previously mentioned, AtPIP1;1, just like other PIP1s, need to heterotetramerize with AtPIP2s to normally localize to the PM (Zelazny et al., 2007; Chevalier et al., 2014; Chevalier and Chaumont, 2015). The localization of PIPs will determine the function of the proteins and even determine the fate of the host cells. Hence, it is imperative to uncover the foremost factors that cause the difference in behavior between PIP1s and PIP2s.

## Results

### AtPIP1;1 Is Unable to Localize in the PM Like AtPIP2;4 When Expressed Alone

**Figure 1:**
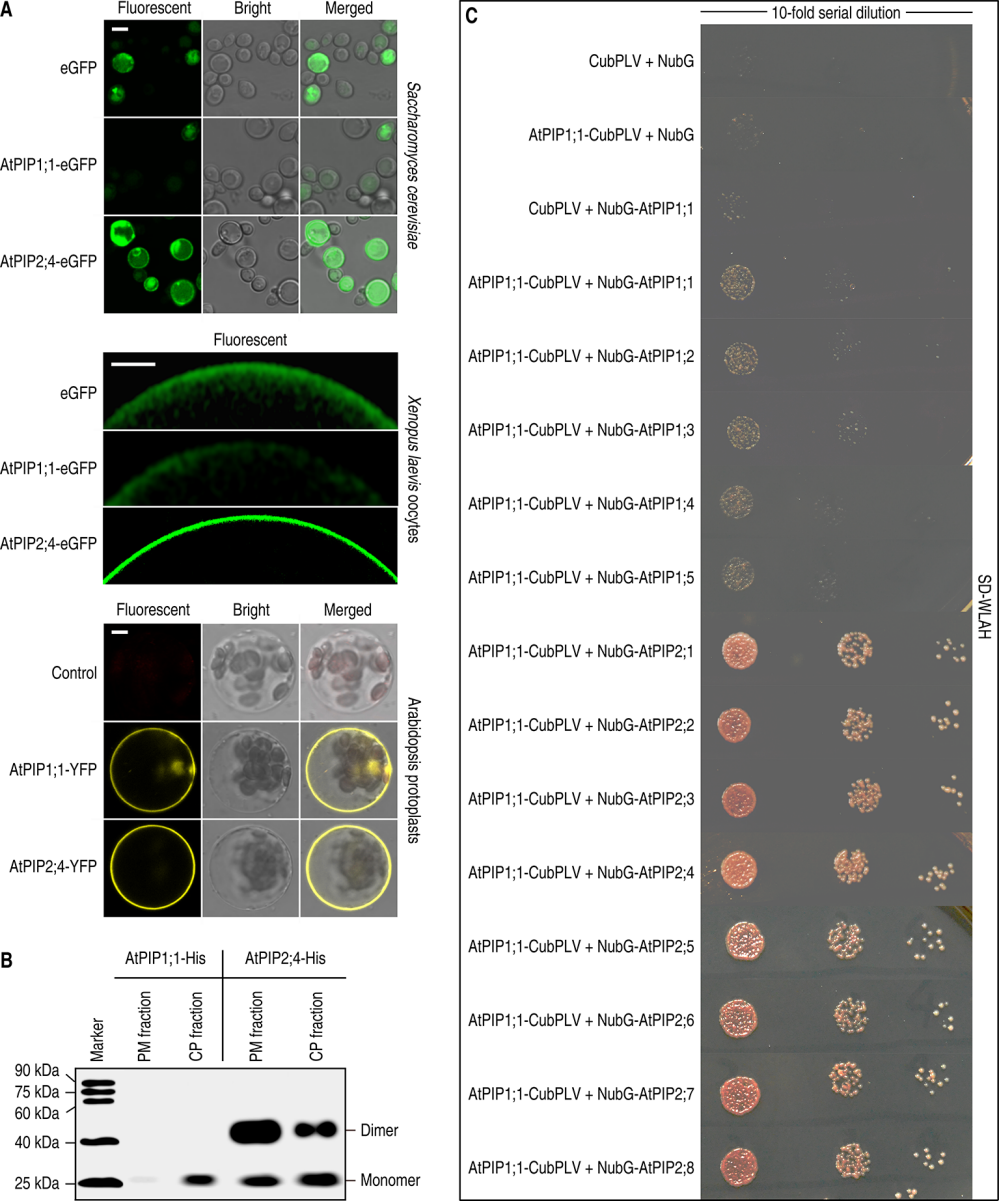
Localization analysis of AtPIP1;1 and AtPIP2;4. **(A)** Subcellular localization of fluorescent proteins fused AtPIP1;1 or AtPIP2;4 expressed in *Saccharomyces cerevisiae* cells (left), *Xenopus laevis* oocytes (middle), and protoplasts of *Arabidopsis thaliana* (right). White scale lines represent the length of 5 μm (upper), 100 μm (middle), and 5 μm (nether). **(B)** Immunoblot of plasma membrane proteins (PM fraction) and intracytoplasmic proteins (including cytosolic proteins and intracellular membrane proteins, CP fraction), extracted from AtPIP1;1-6xHis– or AtPIP2;4-6xHis–transformed yeast cells, with the antibody of anti-6xHis (HT501). **(C)** Interaction between AtPIP1;1 and other AtPIPs using split ubiquitin yeast two hybrid (SUB Y2H) system showing AtPIP1;1 interacted with all the AtPIPs. The transformed yeast cells were series-diluted, and 5 μl of the yeast suspension was spotted on the SD-WLAH plates.

**Figure 2:**
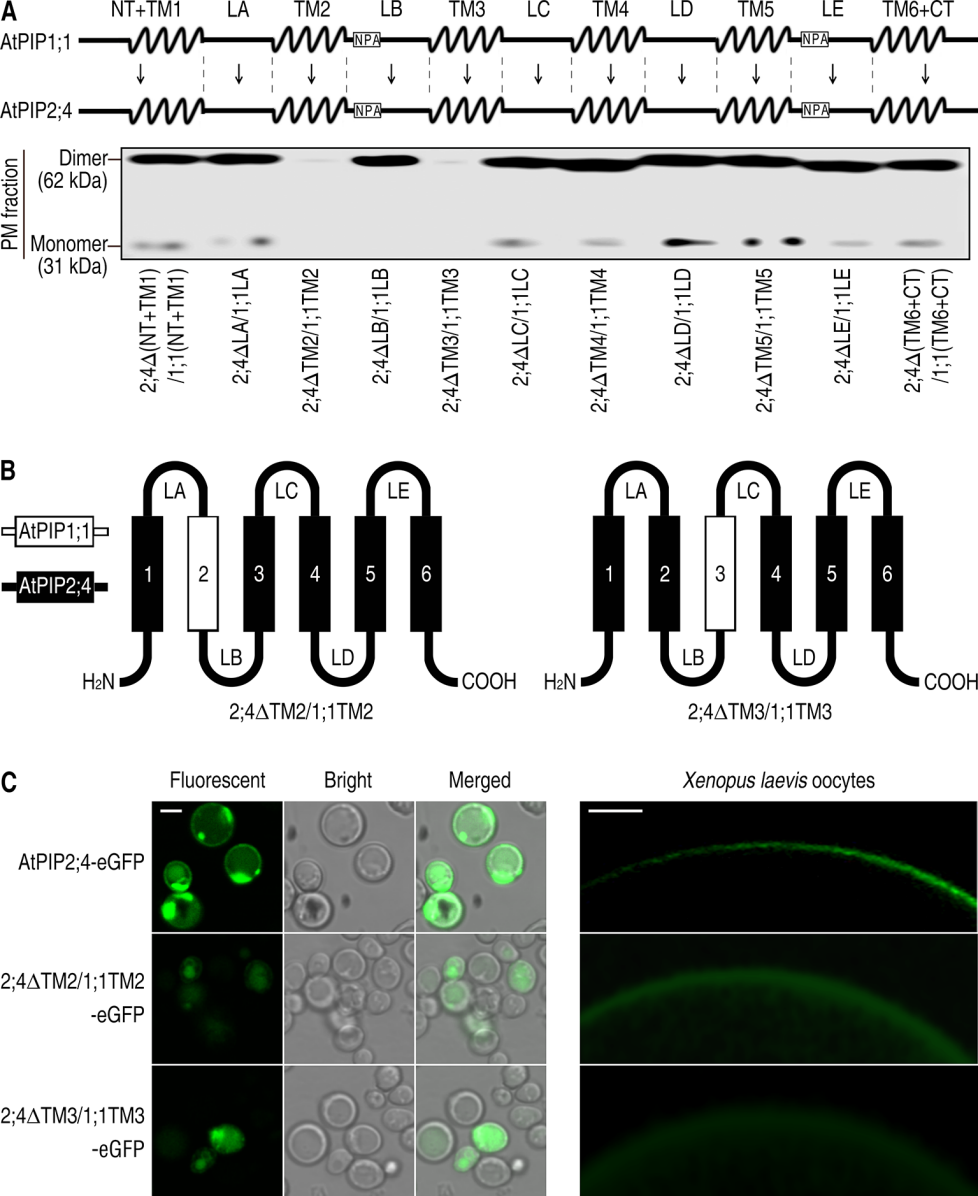
Failed PM targeting of 2;4ΔTM2/1;1TM2 and 2;4ΔTM3/1;1TM3. **(A)** Succinct structure drawing of AtPIP1;1 and AtPIP2;4, which were divided into 11 fragments respectively (upper). Western blot of plasma membrane proteins of the mutants of AtPIP2;4 with different fragments replaced by those of AtPIP1;1 (nether). Membrane proteins were extracted from transformed yeast, and 6xHis antibody was applied. **(B)** Hand-drawn structure of 2;4ΔTM2/1;1TM2 and 2;4ΔTM3/1;1TM3. **(C)** Confocal images of AtPIP2;4-eGFP, 2;4ΔTM2/1;1TM2-eGFP, and 2;4ΔTM3/1;1TM3-eGFP expressing yeast cells (left) and *Xenopus* oocytes (right). White scale lines represent the length of 5 μm (left) and 100 μm (right).

**Figure 3:**
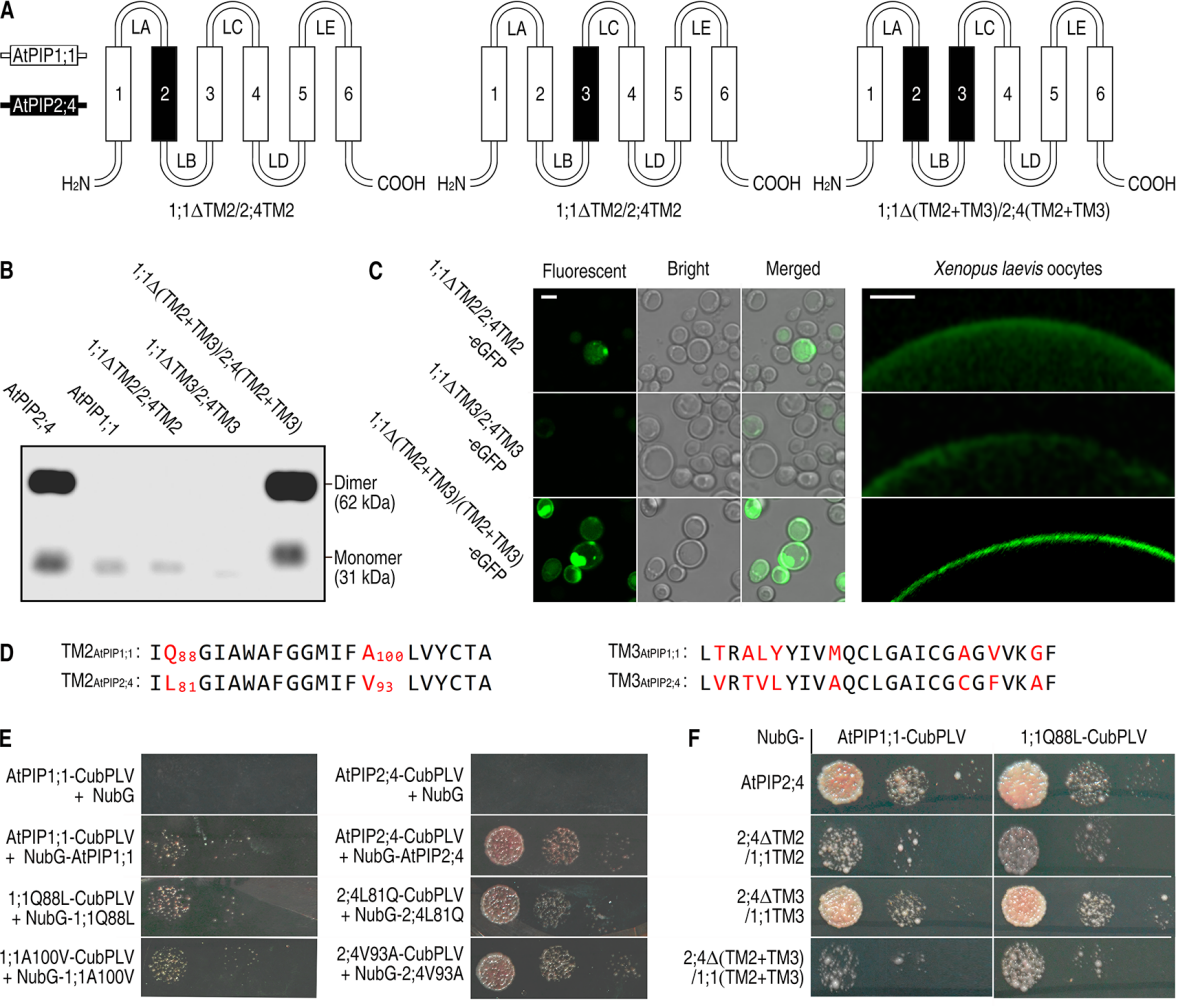
Successful localization of 1;1Δ(TM2+TM3)/2;4(TM2+TM3) in the PM. **(A)** Hand-painted structure of 1;1TM2/2;4TM2, 1;1TM3/2;4TM3, and 1;1Δ(TM2+TM3)/2;4(TM2+TM3). **(B)** Western blot of membrane proteins extracted from AtPIP2;4-, AtPIP1;1-, 1;1ΔTM2/2;4TM2-, 1;1ΔTM3/2;4TM3-, or 1;1Δ(TM2+TM3)/2;4(TM2+TM3)_-_transformed yeast cells using 6xHis antibody. **(C)** Confocal images of 1;1ΔTM2/2;4TM2-eGFP, 1;1ΔTM3/2;4TM3-eGFP, and 1;1Δ(TM2+TM3)/2;4(TM2+TM3)-eGFP expressing yeast cells (left) and *Xenopus* oocytes (right). White scale lines represent the length of 5 μm (left) and 100 μm (right). **(D)** Alignment of transmembrane helix 2 (TM2) and TM3 from AtPIP1;1 and AtPIP2;4. Red letters are the varying residues. Numbers are the sites of the residues. **(E** and **F)** Interaction of mutants of AtPIP1;1 and AtPIP2;4 using SUB Y2H system. The transformed yeast cells were series-diluted, and 5 μl of the yeast suspension was spotted on the SD-WLAH [synthetic dextrose minimal media without W (tryptophan), L (leucine), H (histidine), and A (alanine)] plates.

To investigate the localization of AtPIP1;1 and AtPIP2;4 in different situations, AtPIP1;1 and AtPIP2;4 were expressed in different eukaryotic cells. AtPIP1;1 and AtPIP2;4 were fused with an enhanced green fluorescent protein (eGFP) and transformed into *Saccharomyces cerevisiae* NMY51 cells to examine the localization of each one when expressed alone. Confocal images showed that most of AtPIP2;4-eGFP reached the PM of *S. cerevisiae* cells, while AtPIP1;1-eGFP gathered in the cytoplasm and the fluorescent intensity was significantly lower, which meant most of the expressed proteins might be degraded due to the unstable form and failed PM localization (**Figure 1A** upper). *Xenopus* oocyte expression of cRNA coding AtPIP1;1-eGFP or AtPIP2;4-eGFP gave similar results as that for expression in yeast cells (**Figure 1A** middle). The only difference in oocytes is that the fluorescence of AtPIP2;4 was all at the PM. The immunoblot of the PM proteins and the intracytoplasmic proteins (including cytosolic proteins and intracellular membrane proteins) from AtPIP1;1-6xHis–or AtPIP2;4-6xHis–transformed *S. cerevisiae* cells showed that most of AtPIP2;4-6xHis was in the PM, while AtPIP1;1-6xHis gave a weak signal in the CP fraction and an almost imperceptible signal in the PM fraction (**Figure 1B**). In fact, there was no significant difference in mRNA levels among AtPIP1;1-6xHis, AtPIP2;4-6xHis, and other AtPIP1;1 or AtPIP2;4 variants (**Supplemental Figure 1**). In the oocyte expression experiments, all the proteins could be detected by immunoblotting (**Supplemental Figure 2**). However, in *Xenopus* oocyte expression, AtPIP1;1 or AtPIPs mutants seemed to be soluble in cytosolic space which might be confusing, since PIPs should integrate into membranes (**Figures 1B**, **2C**, **3C**, and **5A**). In fact, the ER cluster massively distributes in cytoplasm of *Xenopus* oocytes (Terasaki et al., 2001). The cytosolic fluorescent signals might be due to the widely distributed ER membrane system in *Xenopus* oocytes.

The examinations in *S. cerevisiae* and *X. laevis* oocytes indicated that AtPIP2;4 can localize in PM when expressed alone, while nearly all the AtPIP1;1 failed to realize it. However, the AtPIP1;1 and AtPIP2;4 fused with a yellow fluorescent protein (YFP) showed transformed protoplasts of *A. thaliana* Col-0 with almost all the fluorescent signal at the PM (**Figure 1A** nether). It was reported that PIP1 could heteroligomerize with PIP2 to realize its PM localization (Zelazny et al., 2007). Therefore, the eight expressed AtPIP2s in wild-type *Arabidopsis* might carry the transiently expressing AtPIP1;1 to the PM. Other than this, the PIPs were promoted by CaMV 35S promoter, which could cause a massive overexpression that might lead to an artifact result. To validate the former assumption, a split ubiquitin yeast two hybrid system (SUB Y2H system) designed for testing interaction of membrane proteins (Obrdlik et al., 2004) was introduced to our study. For testing the strength of their oligomerization, 10-fold series dilution of the transformed yeast was conducted. The results demonstrated that AtPIP1;1 had the capacity of heteroligomerization with all the AtPIP2s (**Figure 1C**). It was most likely that heteroligomerization between AtPIP1;1 and AtPIP2s could provide an explanation for the PM localization of AtPIP1;1 in the AtPIP1;1-YFP transformed protoplasts of *A. thaliana* Col-0. The heteroligomerization and homoligomerization between AtPIP1;1 and AtPIP1s were also found in this test (**Figure 1C**). This kind of heteroligomerization or homoligomerization was also demonstrated for maize ZmPIP1;2 in plant cells by FRET experiments, and in this system, ZmPIP1;2 physically interacts with itself and ZmPIP1;1 (Zelazny et al., 2007). Besides, the AtPIP1;1-CubPLV and NubG-AtPIP2s co-transformed yeast grown much better than AtPIP1;1-CubPLV and NubG-AtPIP1s co-transformed ones on SD-WLAH [synthetic dextrose minimal media without W (tryptophan), L (leucine), H (histidine), and A (alanine)] plates (**Figure 1C**). This result further demonstrated that the affinity between AtPIP1;1 and AtPIP1s was significantly lower than that between AtPIP1;1 and AtPIP2s.

### Switch of TM2 or TM3 From AtPIP1;1 to AtPIP2;4 Disabled PM Targeting

To find out the causes that resulted in localization differences between AtPIP1;1 and AtPIP2;4, we separated them into 11 fragments, based on the structural prediction made with PHYRE2 Protein Fold Recognition Server (**Figure 2A** upper). 2;4ΔTM2/1;1TM2 or 2;4ΔTM3/1;1TM3, which was created by switching TM2 or TM3 from AtPIP1;1 to AtPIP2;4, was rarely detected in the PM when expressed in *S. cerevisiae* or *Xenopus* oocytes (**Figure 2**). A immunoblot analysis was conducted to test the localization of AtPIP1;1 and AtPIP2;4. Immunoblot of the PM proteins, extracted from 2;4ΔTM2/1;1TM2 or 2;4ΔTM3/1;1TM3-transformed *S. cerevisiae* cells, gave almost undetectable signals in their lanes, which meant a failed PM localization (**Figure 2A** nether). 2;4ΔTM2/1;1TM2-eGFP and 2;4ΔTM3/1;1TM3-eGFP were also expressed in *S. cerevisiae* cells or *Xenopus* oocytes to further strengthen the conclusion. As expected, 2;4ΔTM2/1;1TM2-eGFP and 2;4ΔTM3/1;1TM3-eGFP showed fluorescent signal at the cytoplasm (**Figure 2C**). Altogether, TM2 and TM3 played an essential role in PM targeting of AtPIP2;4, while other fragments had insignificant effect on it.

### A Switch of 10 Amino Acids in TM2 and TM3 From AtPIP2;4 to AtPIP1;1 Enables the Localization in PM Like AtPIP2;4

Since the TM2 and TM3 of AtPIP2;4 are necessary for successful localization, are they sufficient to carry AtPIP1 proteins to the PM? To answer that, TM2 and TM3 were switched from AtPIP2;4 to AtPIP1;1, creating three mutants, named 1;1ΔTM2/2;4TM2, 1;1ΔTM3/2;4TM3, and 1;1Δ(TM2+TM3)/2;4(TM2+TM3). 1;1ΔTM2/2;4TM2 and 1;1ΔTM3/2;4TM3 expressed in *S. cerevisiae* cells and *Xenopus* oocytes were mostly apparent in the cytoplasm, while surprisingly, 1;1Δ(TM2+TM3)/2;4(TM2+TM3) showed a strong localization signal in PM just like AtPIP2;4 (**Figure 3**). A western blot of PM proteins, extracted from *S. cerevisiae* cells transformed with 1;1ΔTM2/2;4TM2, 1;1ΔTM3/2;4TM3, or 1;1Δ(TM2+TM3)/2;4(TM2+TM3), was carried out to test PM localization. There were notably weak bands at the lane of 1;1ΔTM2/2;4TM2 and 1;1ΔTM3/2;4TM3, but a very strong signal at the lane of 1;1Δ(TM2+TM3)/2;4(TM2+TM3) (**Figure 3B**). Furthermore, the fluorescent signal of 1;1Δ(TM2+TM3)/2;4(TM2+TM3)-eGFP expressing *S. cerevisiae* cells or *Xenopus* oocytes appeared at the PM sites (**Figure 3C**), which also demonstrated that the TM2 and TM3 of AtPIP2;4 are sufficient to enable AtPIP1;1 mutant to localize in PM like AtPIP2;4. The switch of both TM2 and TM3 from AtPIP2;4 to AtPIP1;1 created a chimeric AQP, which acted just like AtPIP2;4 in localization. Alignment of both TM2 and TM3 of AtPIP1;1 and AtPIP2;4 showed only 10 different residues (**Figure 3D**). In other words, centralized and few residues in TM2 and TM3 dictate the localization of AtPIP1;1 and AtPIP2;4. The previously reported N-terminal DxE motif and C-terminal phosphorylation sites affect their localization, but they are not the decisive factors in localization of AtPIP1;1 and AtPIP2;4.

TM2 is considered to impair the oligomerization of PIPs (Berny et al., 2016; Vajpai et al., 2018). Alignment of TM2 of AtPIP1;1 and AtPIP2;4 showed only two different residues (**Figure 3D**). To test the impact of these residues in oligomerization, SUB Y2H assay was conducted with wild-type and mutated proteins. Here, all the proteins could form homoligomers but with different affinity (**Figure 3E**). AtPIP2;4 or its mutated proteins physically interacted with itself with a strong affinity. In contrast, the interaction of AtPIP1;1 or its mutated proteins had a weak affinity. Interestingly, the interaction of 1;1Q88L (AtPIP1;1 mutant, with glutamine at site of 88 mutated into leucine) was detectably stronger than that of AtPIP1;1 (**Figure 3E**). Meanwhile, the interaction of 2;4L81Q (AtPIP2;4 mutant, with leucine at site of 81 mutated into glutamine) was weaker than that of AtPIP2;4. 1;1A100V (AtPIP1;1 mutant, with alanine at site of 100 mutated into valine) and 2;4V93A (AtPIP2;4 mutant, with valine at site of 100 mutated into alanine) gave no visible difference from their wild-type proteins (**Figure 3E**). In order to test the function of Q88_AtPIP1;1_ in heteroligomerization between AtPIP1;1 and AtPIP2;4, AtPIP1;1 and 1;1Q88L were separately tested in heteroligomerization with AtPIP2;4, 2;4ΔTM2/1;1TM2, 2;4ΔTM3/1;1TM3, and 2;4Δ(TM2+TM3)/1;1(TM2+TM3). In contrast with AtPIP1;1, 1;1Q88L showed a stronger oligomerization with 2;4ΔTM2/1;1TM2 and 2;4Δ(TM2+TM3)/1;1(TM2+TM3) (**Figure 3F**). And the 2;4ΔTM2/1;1TM2 had a significant weaker heteroligomerization with AtPIP1;1 than wild-type AtPIP2;4, which also strengthen the role of TM2 in oligomerization (**Figure 3F**). To quantify above results, the growth rate of SUB Y2H transformants were assayed after a 48 h culture in liquid SD-WLAH medium. It gave a quantitative outcome and indicated the same conclusions as above (**Supplemental Figures 5** and **6**). These results demonstrated that L and Q in TM2 have a significant effect on the oligomerization of AQPs. However, the effect was not enough to determine the localization, since the switching of whole TM2 did not alter localization of AtPIP1;1 and AtPIP2;4.

### 1;1Δ(TM2+TM3)/2;4(TM2+TM3) Shares the Same Capacity With AtPIP2;4 in Water and H_2_O_2_ Transporting

**Figure 4:**
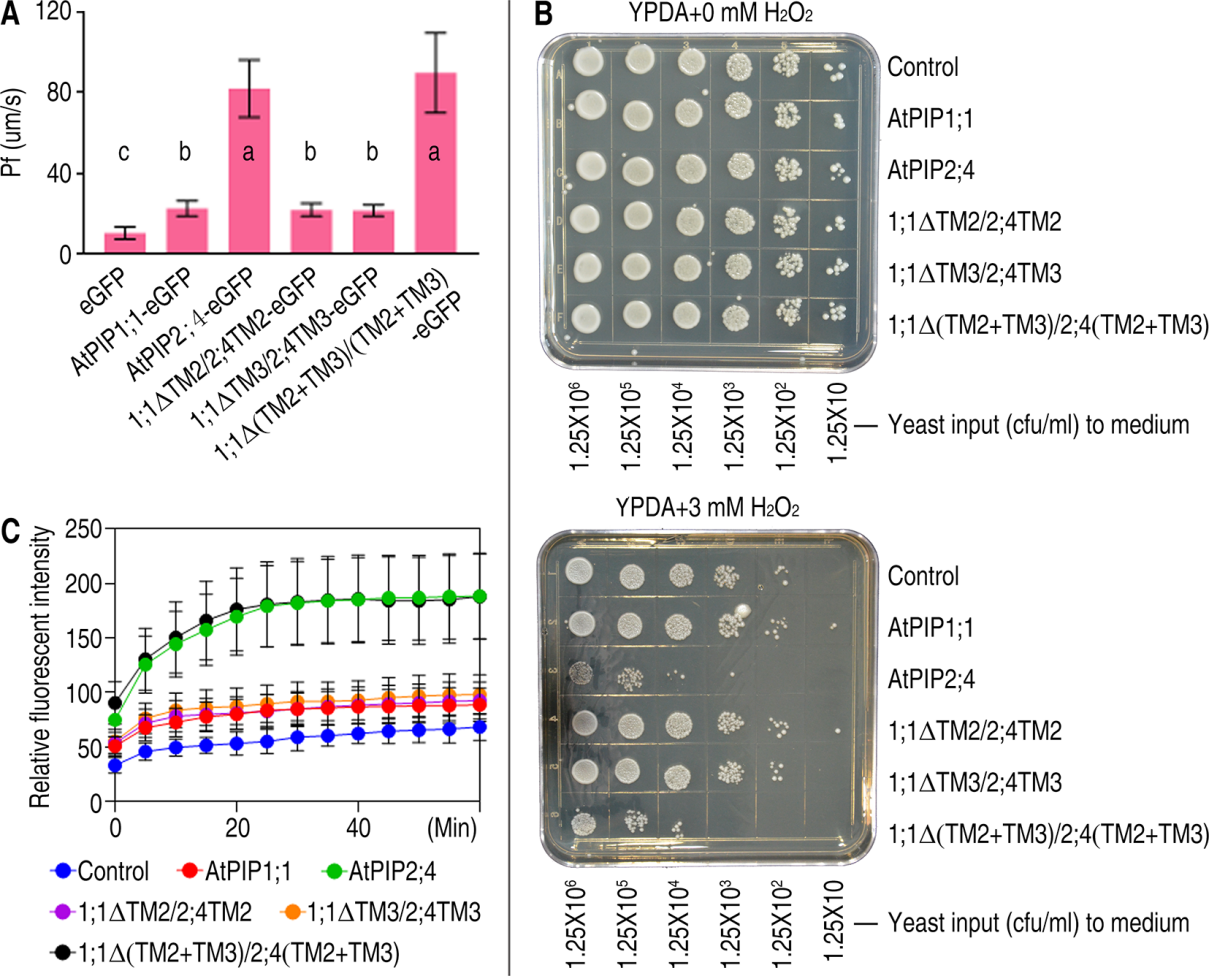
Function analysis of AtPIP1;1, AtPIP2;4, and their mutants. **(A)** Bar graph of Pf values of *Xenopus* oocytes injected with 2 ng cRNA coding eGFP, AtPIP1;1-eGFP, AtPIP2;4-eGFP, 1;1ΔTM2/2;4TM2-eGFP, 1;1ΔTM3/2;4TM3-eGFP, or 1;1Δ(TM2+TM3)/2;4(TM2+TM3)-eGFP. Data shown are the means ± SDs. Different letters indicate significant differences in multiple comparisons of each Pf value using an one-way ANOVA analysis, P < 0.01; for each sample, 10 technical replicates were measured, and the experiments were independently repeated three times. **(B)** Growth status of yeast cells transformed with AtPIP2;4, AtPIP1;1, or their variants spotted on YPDA plates plus 0 or 3 mM H_2_O_2_. **(C)** DCF curves of yeast cells transformed with AtPIP2;4, AtPIP1;1, or their variants with DCF stored and 3 mM H_2_O_2_ added, showing fluorescent signal detected by microplate reader.**** Data are the mean ± SDs. For each sample, triplicates (technical replicates) were measured, and the experiments were independently repeated three times.

PIPs act as PM channels on the premise of its PM localization. Therefore, transport activity of PIPs can also reflect the localization. Based on this, function analysis was carried out. To investigate the water channel transport capacity of 1;1Δ(TM2+TM3)/2;4(TM2+TM3), the membrane osmotic water Pf was determined by injecting cRNA variants coding for AtPIPs to oocytes. The Pf values of AtPIP2;4-eGFP and 1;1Δ(TM2+TM3)/2;4(TM2+TM3)-eGFP are 81.91 ± 14.24 and 89.74 ± 19.80 μm/s respectively. While eGFP and AtPIP1;1-eGFP are 10.27 ± 3.04 and 22.45 ± 3.68 μm/s, respectively (**Figure 4A**). We concluded that AtPIP2;4-eGFP and 1;1Δ(TM2+TM3)/2;4(TM2+TM3)-eGFP are all strong water transporters, and there was no significant difference between AtPIP2;4-eGFP and 1;1Δ(TM2+TM3)/2;4(TM2+TM3)-eGFP in the water transport *Xenopus* oocytes assay at p value <0.05. Compared with AtPIP2;4-eGFP, the Pf of AtPIP1;1-eGFP was only slightly higher than that of eGFP, which indicated that little amount of AtPIP1;1 localized in the PM.

Yeast growth and viability assays were carried out to determine the H_2_O_2_ transport activity. AtPIP2;4-6xHis– and 1;1Δ(TM2+TM3)/2;4(TM2+TM3)-6xHis–transformed *S. cerevisiae* cells showed significant growth deficiency due to the toxicity of excess H_2_O_2_ uptake compared with the control when spotted on the YPDA+ 3 mM H_2_O_2_ plates (**Figure 4B**). A cytoplastic H_2_O_2_ fluorescent dye named H2DCFDA was applied to evaluate H_2_O_2_ accumulation in *S. cerevisiae* cells. H_2_O_2_ was added to the suspension of yeast cells after H2DCFDA installed in cytoplasm. The fluorescence intensity of AtPIP2;4- and 1;1Δ(TM2+TM3)/2;4(TM2+TM3)-transformed cells are all significantly stronger than control (**Figure 4C**), which implied 1;1Δ(TM2+TM3)/2;4(TM2+TM3) has similar ability to regulate H_2_O_2_ across the membrane. And like the results of water transport assay, H_2_O_2_ transport activity of AtPIP1;1-transformed yeast cells was slightly stronger than the control (**Figure 4C**). This slight difference could not even be detected by the yeast growth and viability assay. Previous studies show PIP1s own an original capacity of transporting solutes (Fetter et al., 2004; Bienert et al., 2018). The switch of TM2 and TM3 from AtPIP2;4 to AtPIP1;1 did not alter the original potential of AtPIP1;1 as a water and H_2_O_2_ transporter, but truly enabled the localization in PM.

### Other AtPIP1 Variants, With (TM2+TM3)_AtPIP2;4_ Fragments, Can Also Be Localized in PM Spontaneously and Play an Inherent Role in Transporting Solutes

**Figure 5:**
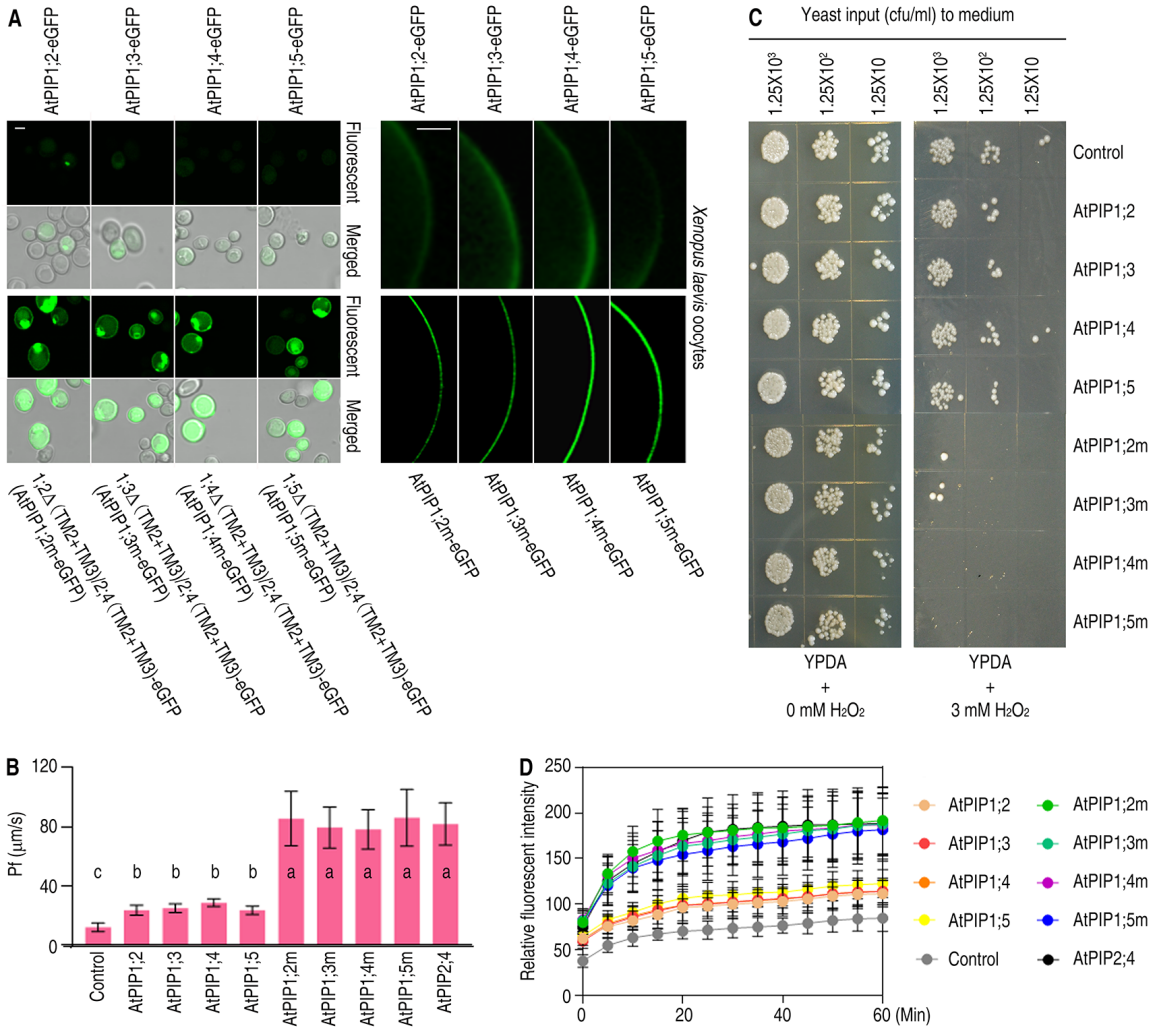
Subcellular localization and function analysis of AtPIP1;2, AtPIP1;3, AtPIP1;4, AtPIP1;5, and their mutants. **(A)** Confocal images of yeast cells (left) and *Xenopus* oocytes (right), which were expressing AtPIP1;2-eGFP, AtPIP1;3-eGFP, AtPIP1;4-eGFP, AtPIP1;5-eGFP, and their eGFP-fused mutants_._ White scale lines represent the length of 5 μm (left) and 100 μm (right). **(B)** Bar graph of Pf values of *Xenopus* oocytes injected with 2 ng cRNA coding eGFP, AtPIP1;2-eGFP, AtPIP1;3-eGFP, AtPIP1;4-eGFP, AtPIP1;5-eGFP, and their eGFP-fused mutants. On bar graphs, different letters indicate significant differences in multiple comparisons of each Pf values using an one-way ANOVA analysis, P < 0.01; for each sample, 10 technical replicates were measured, and the experiments were independently repeated three times. **(C)** Growth status of yeast cells transformed with AtPIP1;2, AtPIP1;3, AtPIP1;4, AtPIP1;5, or their mutants spotted on YPDA plates plus 0 or 3 mM H_2_O_2_. **(D)** DCF curves of AtPIP1;2, AtPIP1;3, AtPIP1;4, AtPIP1;5, or their mutant-transformed yeast cells with DCF stored and 3 mM H_2_O_2_ added, showing fluorescent signal detected by microplate reader. Data are the mean ± SDs. For each sample, triplicates (technical replicates) were measured, and the experiments were independently repeated three times.

It is similar of AtPIP1 isoforms in amino acid sequences and structures, which makes it a legitimate inference for (TM2+TM3)_AtPIP2;4_ to carry other AtPIP1s to PM. To fully confirm the effect of TM2 and TM3 on localization, all the other AtPIP1 isoforms, including AtPIP1;2, AtPIP1;3, AtPIP1;4, AtPIP1;5, and their (TM2+TM3) switched mutants, were expressed in yeast cells and *Xenopus* oocytes. Fluorescent images showed TM2- and TM3-switched AtPIP1 variants accomplished PM localization in yeast cells and *Xenopus* oocytes, just like 1;1Δ(TM2+TM3)/2;4(TM2+TM3) (**Figure 5A**). It is concluded that (TM2+TM3)_AtPIP2;4_ is sufficient for all the AtPIP1s to reach PM.

For a further comprehension of the importance of the correct localization of AtPIP1s, H_2_O and H_2_O_2_ transport assays were carried out. The capacity of transporting H_2_O was tested, since water channel was the inherent function of AQPs. Pf values were measured based on the water swelling of the *Xenopus* oocytes with AtPIP1 variants expressed in. The Pf values of AtPIP1;2, AtPIP1;3, AtPIP1;4, and AtPIP1;5 showed a slight difference from the control while the Pf values of (TM2+TM3)_AtPIP2;4_ replaced AtPIP1 variants were significantly higher than the control and the wild-type AtPIP1s, just like AtPIP2,4 (**Figure 5B**). To test the capacity of transporting H_2_O_2_, yeast growth and viability assays were conducted and showed AtPIP1 mutant-transformed yeast cells grew more slowly compared with wild-type AtPIP1 isoforms and the control (**Figure 5C**). DCF fluorescence assay also showed that AtPIP1 mutant-transformed yeast cells had stronger fluorescence signal compared with wild-type AtPIP1 isoforms and the control, which meant a faster uptake of H_2_O_2_ (**Figure 5D**). Altogether, the AtPIP1 mutants, with TM2 and TM3 replaced by those of AtPIP2;4, had an inherent capacity to transport H_2_O and H_2_O_2_ when they spontaneously reached the PM, like the performance of 1;1Δ(TM2+TM3)/2;4(TM2+TM3).

## Discussion

### TM2 and TM3 Play Different Roles in Localization

**Figure 6:**
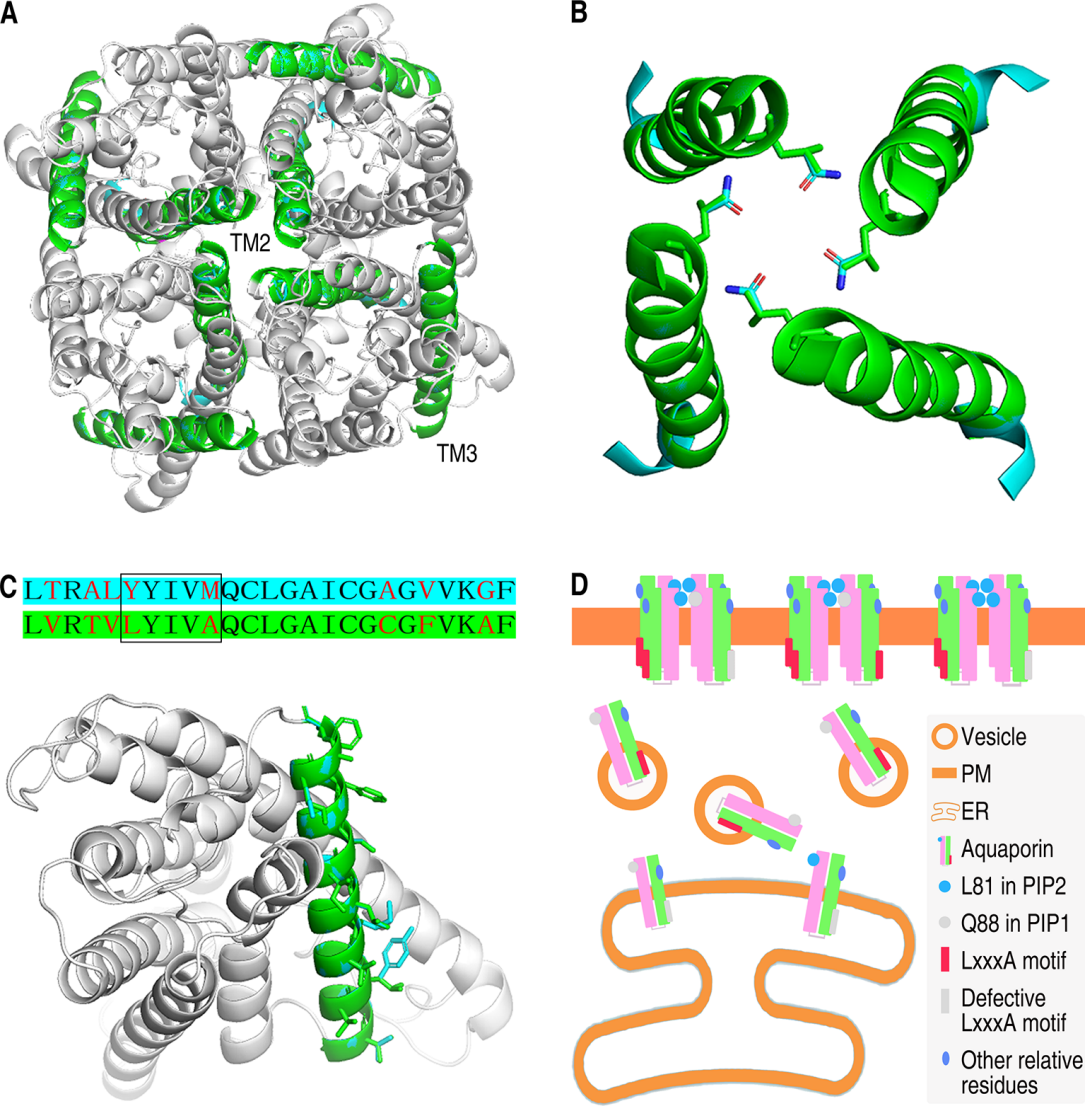
Structure analysis and localization working model of AtPIP1;1 and AtPIP2;4. **(A)** Tetrameric structure of AtPIP2;4 with AtPIP1;1 aligned in. TM2s and TM3s are highlighted with cyan (AtPIP1;1) and green (AtPIP2;4). **(B)** Structure of four TM2s from AtPIP2;4 tetramer (green) with those from AtPIP1;1 (cyan) aligned in. The Q residue and the corresponding L residue are in the form of sticks. Red in the sticks represents oxygen atom, while blue represents nitrogen atom. **(C)** Sequences alignment of TM3 region of AtPIP1;1 with cyan background and AtPIP2;4 with green background (upper) and structure of AtPIP1;1 and AtPIP2;4 with TM3 highlighted in cyan (AtPIP1;1) or green (AtPIP2;4) (nether). Red letters are the varying residues, and LxxxA region is in the square frame (upper). All outfacing residues are in the form of sticks (nether). **(D)** Working model of the subcellular localization of plasma membrane intrinsic proteins. Other relative residues are some of the different residues except the L81 and LxxxA motif in TM2 and TM3 between PIP1s and PIP2s that might also take part in localization of PIPs.

TM2 and TM3 are important segments for the localization of PIPs. For further understanding about the role of TM2 and TM3 in determining subcellular localization of PIPs, structural models were set up based on the previous AQP structure models. AtPIP1;1 and AtPIP2;4, being the focus of this study and the representatives of AtPIP1 group and AtPIP2 group, were logically chosen for the structural analysis. We set up a tetramer model of AtPIP2;4, which compromises four predicted monomers (**Figure 6A**). Afterward, four monomers of AtPIP1;1 were aligned with this tetramer of AtPIP2;4, and from the model, we found that TM2 is in inside the tetramer, while TM3 faces the outside. Heterotetramer of AtPIP1;1 and AtPIP2;4 is far more likely to share the same framework as that of the AtPIP2;4 homotetramer to keep the tetramer in a stable form. The physical position of TM2 indicates its roles in oligomerization. The TM2 of AtPIP1s and AtPIP2s is highly conserved respectively, especially the residues extending outward (**Supplemental Figure 3**), which could be an explanation that the interaction of AtPIP1;1 with its AtPIP2 partners was similar (**Figure 1C** and **Supplemental Figure 4**). Besides, TM2s of AtPIP1;1 and AtPIP2;4 were extracted from the tetramer model with Q88 and L81 in the stick form (**Figure 6B**). TM3s of AtPIP1;1 and AtPIP2;4 were also highlighted in the superimposed monomers, and the outfacing residues are in stick forms (**Figure 6C**). Due to the difference in physical position of the TM2 and TM3 and chemical property of the residues, TM2 and TM3 may play different roles in localization.

PM-targeting PIPs undergo ER–Golgi trafficking and finally reach the PM. Wide studies on localization of PIPs have been done. But the localization of PIPs is still not fully uncovered. When PIP1s were expressed in plant cells, almost all the PIP1s localized in the PM (**Figure 1A** nether). This phenomenon could be explained by the heterotetramerization between PIPs and PIP2s. PIP1s lack a PM ER secreting signal. But the heterotetramerization with PIP2s makes up for the defect of PIP1 group in ER–Golgi trafficking and even drives PIP1 to the PM. Therefore, whether the oligomerization happens between PIP1s and PIPI2s tremendously impacts the localization of PIP1s. The interfaces between the monomers in the tetrameric ZmPIP1;2 contains TM1–TM2 and TM4–TM5 (Vajpai et al., 2018). Residual amino acids in TM2 could play dominant roles in the tetramerization.

Several motifs and residual amino acids are demonstrated to importantly impact the localization. Among them, LxxxA motif in TM3 plays a vital role in localization process of PIPs. LxxxA motif may act as an ER secreting signal (Chevalier et al., 2014). PIP1s, lacking LxxxA motif, is thus expected to be detained in ER. However, the detaining does not happen to all the PIP1s molecules, since certain amount of PIP1s do localize in the PM in *Xenopus* oocytes (Chevalier et al., 2014). This is also demonstrated by our results. The mechanism in this process is not well interpreted.

### TM2 May Affect the Tetramerization at L81

As previously referred, alignment of TM2 fragments from AtPIP1;1 and AtPIP2;4 showed only two different residue sites (**Figure 3D**). Glutamine is an uncharged polar amino acid, while leucine is a non-polar hydrophobic amino acid. Theoretically, embedding the hydrophobic side groups exposed to the outside of a protein is the motive power of the oligomerization. Here, if the L81 at the central position of the tetramer was replaced to Q88, it would be an impediment to the tetramerization due to the hydrophilic side group (**Figure 6B**). In interaction test, leucine in TM2 was also demonstrated to affect the oligomerization of PIPs. In maize, when glutamine in TM2 of ZmPIP1;2 was mutated into leucine, the dimeric form was more abundant than monomeric form in western blot experiment, also suggesting leucine could contribute to oligomerization (Berny et al., 2016). Furthermore, the glutamine and leucine at the corresponding site are totally conservative in AtPIP1 group and AtPIP2 group (**Supplemental Figure 3**). As one can imagine, this site in TM2 is far more likely to be an important difference between AtPIP1s and AtPIP2s.

### Residues in TM3 Could Affect the ER–Golgi–PM Trafficking, Especially the LxxxA Motif

TM3, reported previously as an important domain of PIPs to affect the localization, contains an LxxxA motif in ZmPIP2;5 which is considered to be a secreting signal (Chevalier et al., 2014). Our work enforces the role of TM3 in localization. Alignment of TM3 fragments of AtPIP1;1 and AtPIP2;4 showed eight different residue sites, including the LxxxA motif region (**Figure 5C** upper). In TM3 of AtPIP1;1, the LxxxA region becomes YxxxM (Y, tyrosine; M, methionine), giving AtPIP1;1 a significant difference. In AtPIP2 group, AtPIP2;1, AtPIP2;2, AtPIP2;3, and AtPIP2;4 have LxxxA motif, others with MxxxA, SxxxA (S, serine), GxxxA (G, glycine), or AxxxA, while AtPIP1 isoforms only have YxxxM or FxxxM (F, phenylalanine) motifs (**Supplemental Figure 3**). Y and F, the first residue of this region in AtPIP1 isoforms, are typical aromatic amino acids, but the corresponding L, M, S, G, A residues in AtPIP2 isoforms are not. The last residue in this region is M in AtPIP1 group, but an A in AtPIP2 isoforms. Although they are all hydrophobic residues, a longer side group and an additional sulfur should give M another image. In summary, the distinction between AtPIP1 group and AtPIP2 group in the LxxxA region could make a huge difference in their ER–Golgi–PM trafficking.

### Other Residues in TM2 and TM3 Are Also Important for Localization

It is not concluded that the residues except L81 and LxxxA in TM2 and TM3 do not impact the localization of PIPs. In fact, 1;1Δ(Q88L+LxxxA), which has the L and LxxxA motif, cannot localize into the PM like AtPIP2;4 (**Supplemental Figure 7**). In TM2, the other couple of different residues, corresponding to valine and alanine, are similar with each other in side groups. And the relative position of them is completely different from that of L81 and Q88. Therefore, it might affect the localization by a method other than tetramerization. Besides, the other different residues in TM3 between AtPIP1 and AtPIP2 may also play some role in the localization. The orientation, length, and hydrophilicity of the side groups of these residues show a large difference that might determine the affinity to the phospholipid membrane, based on TM3 being outfacing in the tetramer structure (**Figure 6C** nether).

### Working Model for the Localization of PIPs

Overall, TM2 and TM3 determine which AtPIPs is able to be released from ER to Golgi and finally localize in the PM. AtPIP1 isoforms cannot completely implement the ER–Golgi–PM trafficking when expressed alone due to the defective homotetramerization and trafficking signal. A heterotetramerization with AtPIP2 isoforms will provide AtPIP1s with a stable tetrameric form and a strong ER secreting signal, which carry the AtPIP1 molecule to the PM. Besides, some other relative residues in TM2 and TM3 will also influence the localization of PIPs in some other way.

## Materials and Methods

### Cloning and Generating

cDNAs of *AtPIP1;1*, *AtPIP1;2*, *AtPIP1;3*, *AtPIP1;4*, *AtPIP1;5*, and *AtPIP2;4* were amplified from RNA templates of 3-week-old *A. thaliana* ecotype Col-0. Mutants of *AtPIP1;1*, *AtPIP1;2*, *AtPIP1;3*, *AtPIP1;4*, *AtPIP1;5*, and *AtPIP2;4* were generated through fusion polymerase chain reaction (fusion-PCR, Szewczyk et al., 2006) or single-residue mutation method. For fusion-PCR, two or more fragments of *AtPIP1;1*, *AtPIP1;2*, *AtPIP1;3*, *AtPIP1;4*, *AtPIP1;5*, or *AtPIP2;4* were amplified with homologous recombination arms which were paired with complementary targeting fragments. The complementary pairing fragments were incubated together with DNA polymerase for 12 PCR cycles. Then the full-length primers of the mutated AQPs were added in the products to conduct a PCR procedure. Products were purified with the Cycle Pure Kit (D6492-02). For single-residue mutation, pairs of partially complementary primers were designed at the sites of the targeting residues. The whole vectors with wild-type *AtPIPs* genes inserted were amplified with the primers. After digesting the template vectors by *Dpn* I, the purified products were transformed into DH5α. Positive transformants would be the single-residue mutants.

### Localization Assay in Yeast

cDNAs fused with *eGFP* were constructed in *S. cerevisiae* expression vector, pYES2, with restriction enzyme combinations of *Hin*d III and *Eco*R I or *Kpn* I and *Eco*R I. The resulting plasmids were transformed into *S. cerevisiae* NMY51 competent cells in transformation solution (0.1 M LiCl, 30% w/v PEG4000). The positive transformants were cultured in SD-Ura (SD without uracil) media with 2% w/v galactose overnight at 30°C and harvested in phosphate buffer solution (PBS; 0.2 mM, pH 7.4). Cells were observed between 495 and 520 nm using 488 nm argon-ion laser excitation with a Zeiss LSM700 laser scanning confocal microscope.

### Protein Localization Assay in *Xenopus* Oocytes

The use of *Xenopus* oocytes was evaluated and approved by the ethics committee of Nanjing Agricultural University and carried out in accordance with the guidelines provided by this committee.

A Kozak sequence, GCCACC, was placed in front of initiator codon ATG to enhance the translational efficiency (Raif et al., 2010). Kozak sequence containing cDNA-fused *eGFP* were constructed into pGH19 with restriction enzyme combination of *Eco*R I and *Xba* I. The generated plasmids were linearized with *Not* I restriction enzyme and purified in RNase free water. One microgram for each linearized DNA was applied for a *in vitro* transcription using RiboMAX™ Large Scale RNA Production Systems-T7 (P1300). Two nanograms resulting cRNA (without DNA) was injected into the Dumont stage V *Xenopus* oocytes. The generated oocytes were cultured in sterile ND96 solution (93.5 mM NaCl, 2 mM KCl, 1.8 mM CaCl2, 2 mM MgCl2, 5 mM Hepes, pH 7.50) with penicillin and streptomycin added at 18°C for 36 h. Oocytes were fixed with paraformaldehyde and then sectioned into halves. Oocytes were finally observed between 495 and 520 nm using 488 nm argon-ion laser excitation with a Zeiss LSM700 laser scanning confocal microscope.

### Pf Examination

The osmotic water permeability coefficient (Pf) value was determined based on the expression of AQPs in *Xenopus* oocytes. Oocytes were transferred from ND96 solution to a fivefold diluted ND96 solution after 2 days’ culture at 18°C. The swelling of the oocytes was monitored by a stereomicroscope linked to a black-and-white digital camera. Images were captured at 5 s intervals for 30 cycles, and the diameter of the oocytes for every cycle was measured for the calculation of the Pf values. The Pf was calculated using the equation Pf = V_0_[d(V/V_0_)/dt]/[S × V_w_(Osm_in_ − Osm_out_)], in which the initial volume (V_0_) equals 9 × 10^−4^ cm^3^, the initial oocyte surface area is 0.045 cm^2^, and the molar volume of water (V_w_) is 18 cm^3^/mol (Preston et al., 1992; Fetter et al., 2004).

### Purification of Proteins

The transformed yeast cells were pre-cultured in a shaker at 180 rpm and 30°C overnight. The culture of yeast was started with an OD600 of 0.1 adjusted with the pre-cultured yeast suspension. Cells were harvested after 24 h and then broken by vortex with 0.5 mm glass beads in 0.3 M sucrose containing 0.1 mM phenylmethanesulfonyl fluoride and 0.1 mM leupeptin. Cell debris was removed by a 15,000 × g spin at 4°C for 2 × 20 min. The PM fractions were prepared by differential centrifugation as previously described (Marinelli et al., 1997; García et al., 2001). Briefly, the PM fraction was obtained by centrifugation at 200,000 × g for 1 h on a discontinuous 1.3 M sucrose gradient. The PM band was isolated and subsequently mixed with 200 mM NaCl, 50 mM Tris pH 8, 2% n-dodecyl-N,N-dimethylamine-N-oxide (PM fraction). The left fraction was cytoplastic proteins and intracellular membranes (CP fraction). The PM fraction and CP fraction were then applied to western blot assay.

To extract the protein of *Xenopus* oocytes, cRNA injected oocytes were broken byvortex at 4°C for 5 min and homogenized in PBS with 2% SDS.

### Localization Assay in Protoplast

cDNAs were constructed in a re-modified plant expression vector pCAMBIA 1300 35S::*YFP::*poly(A), with restriction enzyme combinations of *Kpn* I and *Xba* I or *Sac* I and *Xba* I. As a result, cDNAs ligased with *YFP* sequence were inserted between CaMV 35S promoter and CaMV poly(A) signal sequence, thus forming a highly efficient expression system. Protoplast isolation and transient expression were conducted based on the previous study (Hoffman et al., 1994). Leaves of *Arabidopsis* were cut into 0.5 × 5 mm strips and thrown in enzyme solution (1% w/v cellulase R10, 0.2% w/v Macerozyme R10, 0.4M mannitol, 20 mM KCl, 20 mM MES, pH 5.7, 10 mM CaCl_2_, 0.1% BSA). After a 30 min vacuum treatment and a 2 h incubation at 40 rpm and 23°C, protoplast was released in the solution. The protoplast containing solution was added with W5 solution (154 mM NaCl, 125 mM CaCl_2_, 5 mM KCl, 2 mM MES, pH 5.7) to stop the enzyme reaction, along with a 0.1 mm aperture nylon mesh filtration. Protoplast was spun down at 100 rpm and 4°C for 5 min, washed with W5 solution, and finally resuspended in MMG solution (0.4 M mannitol, 15 mM MgCl_2_, 4 mM MES, pH 5.7). The obtained protoplast was incubated with corresponding plasmids in PEG solution (40% w/v PEG, 0.2 M mannitol, 100 mM CaCl_2_) at 23°C for 15 min. The reaction was stopped by adding equal volume of W5 solution. Transformed protoplast was obtained by 100 rpm spin at 4°C for 5 min, resuspended in W5 solution, and cultured in darkness at 23°C for 16 h. Resulting protoplast was observed between 520 and 535 nm using 514 nm argon-ion laser excitation with a Zeiss LSM700 laser scanning confocal microscope.

### Proteins Interaction Assay Using SUB Yeast Two Hybrid System

This experiment was carried out based on the previous study (Obrdlik et al., 2004). Briefly, the cDNAs of *AtPIP1;1* and other *AtPIP*s were constructed in pMetYCgate and pXNgate respectively. The combination of pMetYCgate-*AtPIP1;1* and pXNgate-*AtPIP* were transformed into *S. cerevisiae* NMY51. Transformants were spread on SD-WL (SD without W and L), SD-WLH (SD without W, L, and H), and SD-WLAH plates. Positive transformants were 10-fold serially diluted and spotted on SD-WLAH plates and incubated at 30°C for 2 days. And the positive transformants with original OD_600__=_ 0.01 were cultured in liquid SD-WLAH medium at 200 rpm, 30°C for 2 days. The final OD_600_ of resulting yeast culture was then measured by UV-VIS spectrophotometer (UV-2700).

### Yeast Growth and Viability Assay

cDNAs fused with 6xHis tag were ligated to *S. cerevisiae* expression vector pYES2, with restriction enzyme combinations of *Hin*d III and *Eco*R I or *Kpn* I and *Eco*R I. The resulting plasmids were transformed into *S. cerevisiae* strain NMY51, competent cells in transformation solution (0.1 M LiCl, 30% w/v PEG4000). And the positive transformants were cultured in SD-Ura media with 2% w/v galactose overnight at 30°C and harvested in PBS (0.2 mM, pH 7.4). The cell pellets were washed with PBS and diluted to a normalized cell density of OD_600__=_ 1. Three repeating 10-fold serial dilutions were prepared from the normalized suspension for each transformant. Six dilutions, 10 μl of each, from every transformant were spotted on the YPD agar plates with or without 3mM H_2_O_2_. The yeast was incubated at 30°C for 3–4 days.

### DCF Fluorescence Assay

The transformants in yeast growth and viability assays were applied to DCF fluorescent assay. The yeast cells were resuspended in PBS along with an addition of H2DCFDA (D399) at a final concentration of 10 μM (Saito et al., 2003; Tian et al., 2016). After an incubation for 30 min at 30°C, yeast cells were washed twice by PBS and resuspended in PBS with H_2_O_2_ at a final concentration of 3 mM. Fluorescence densities in transformed yeast cells were evaluated with a SpectraMax M5 96 microplate reader to estimate the H_2_O_2_-transporting capacity of AtPIP1;1, AtPIP2;4, and variant isoforms of them.

### RT-qPCR

RNA of transformed yeast cells was extracted using PureLink™ RNA Mini Kit (12183025), and cDNAs were obtained from reverse transcription PCR. The template cDNAs were mixed with RT-qPCR primers of AtPIP1;1, AtPIP1;2, AtPIP1;3, AtPIP1;4, AtPIP1;5, AtPIP2;4, and the yeast endogenous reference gene, *glyceraldehyde-3-phosphate dehydrogenase 1* (*TDH1*), to conduct RT-qPCR assay using SYBR^®^ Premix Ex Taq™ II kit (RR820A).

### Structure Analysis

The structures of AtPIP1;1 and AtPIP2;4 were predicted and set up by the PHYRE2 Protein Fold Recognition Server. The tetramer model of AtPIP2;4 was set up based on the model of SoPIP2;1 tetramer (PDB ID 4IA4) using PyMOL software. Four AtPIP1;1 monomers were aligned in the model of AtPIP2;4 tetramer that formed an AtPIP1;1 tetramer.

## Data Availability Statement

All datasets generated for this study are included in the article/**Supplementary Material**.

## Ethics Statement

The use of Xenopus oocytes was evaluated and approved by the ethics committee of Nanjing Agricultural University and carried out in accordance with the guidelines provided by this committee.

## Author Contributions

HD and DS conceived the project and supervised the experiments. HW, LZ, YT, and ZW performed most of the experiments. HW analyzed the data and wrote the article with contributions of all the authors. HD supervised the writing and agrees to serve as the author responsible for contact and ensures communication.

## Funding

This work was supported by the Natural Science Foundation of Jiangsu Province (grant number BK20150668) to DS, China National Key Research and Development Plan (2017YFD0200901) to HD, and Natural Science Foundation of China (31772247) to HD.

## Conflict of Interest

The authors declare that the research was conducted in the absence of any commercial or financial relationships that could be construed as a potential conflict of interest.
