# Supplementary material for: Transmembrane Helices 2 and 3 Determine the Localization of Plasma Membrane Intrinsic Proteins in Eukaryotic Cells
Source: Front Plant Sci. 2020 Jan 10;10:1671. doi: 10.3389/fpls.2019.01671 (PMC6966961; doi:10.3389/fpls.2019.01671)
Supplement: Supplementary file 1 [file DataSheet_1.doc]

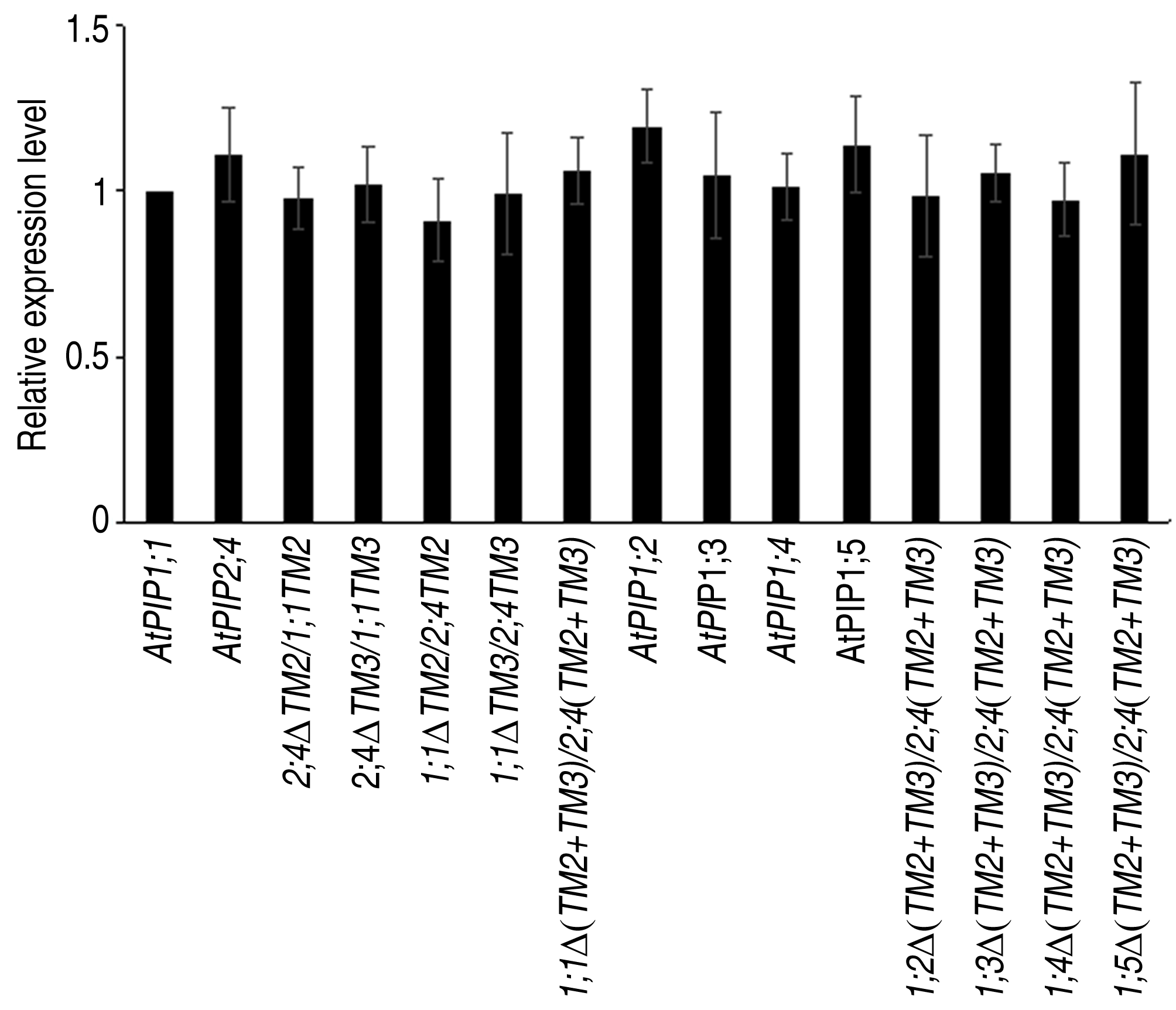


**Supplemental Figure 1.** Relative expression level (amount of mRNA) of *AtPIP1s*, *AtPIP2;4* and their mutants in transformed *S. cerevisiae* NMY51 estimated by a RT-qPCR.


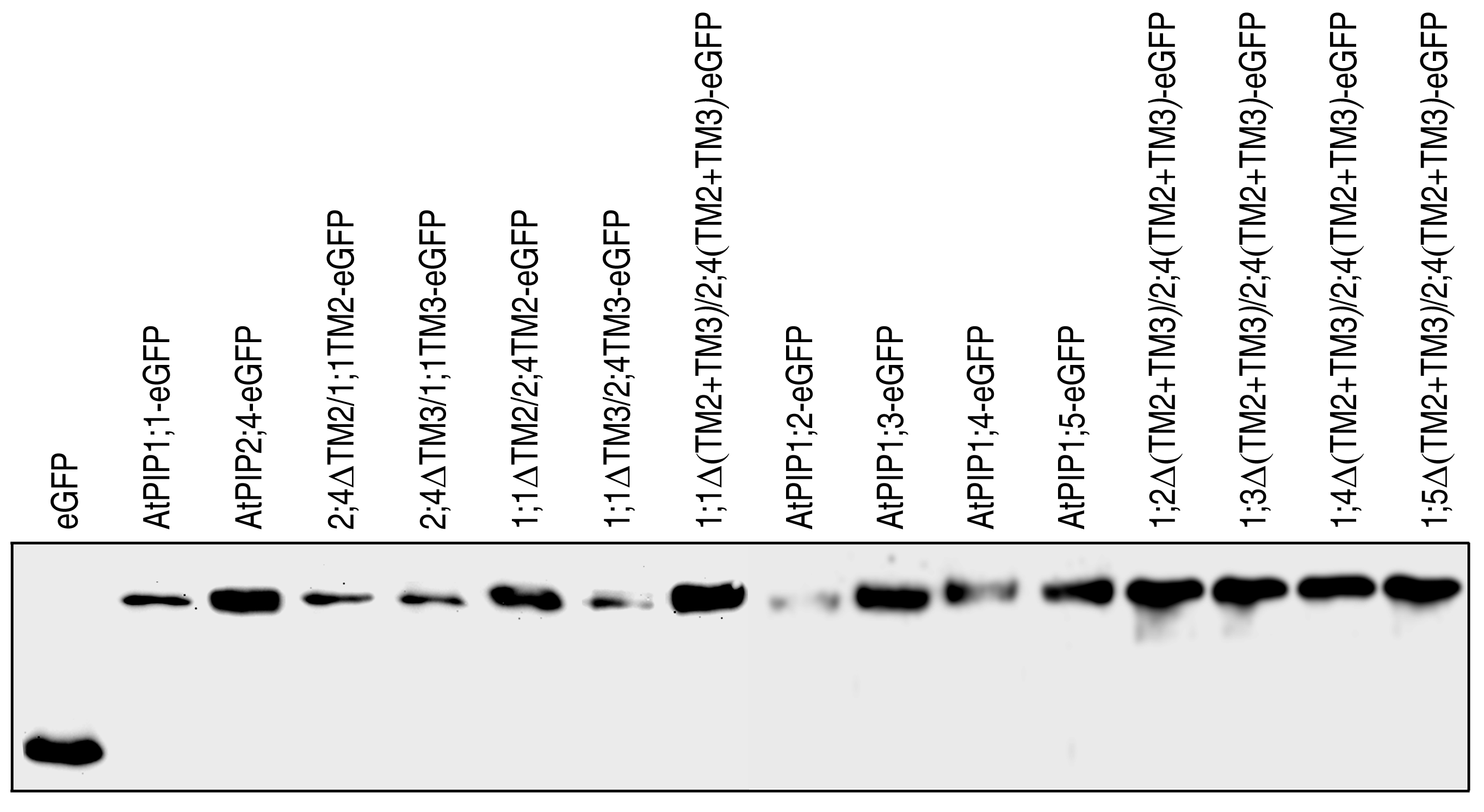


**Supplemental Figure 2:** Immunoblot of proteins extracted from Xenopus oocytes injected with cRNA coding eGFP, AtPIP1;1-eGFP, AtPIP2;4-eGFP and their mutants using anti-GFP antibody.


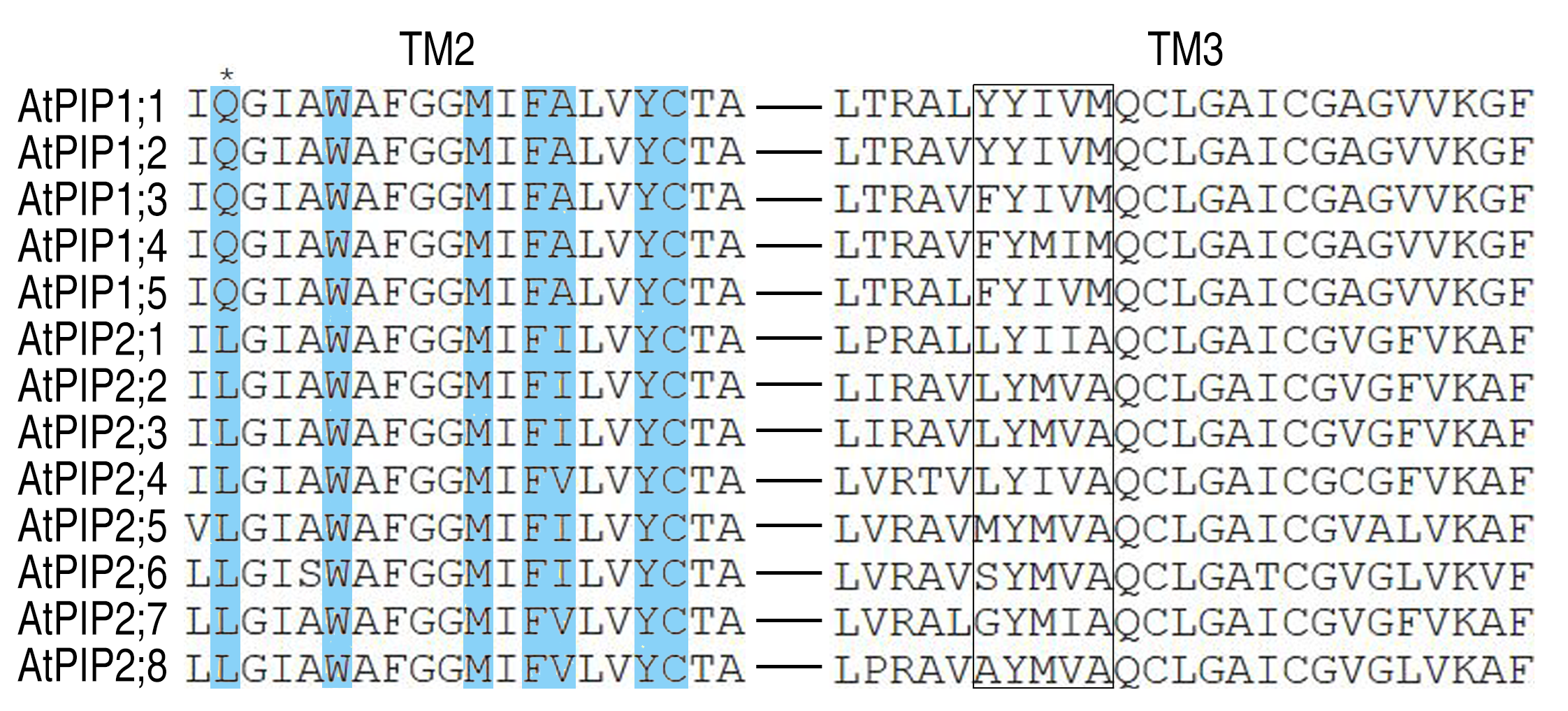


**Supplemental Figure 3:** Alignment of both TM2 and TM3 among all the AtPIPs. Quincunx points the Q88 and L81 residues; Residues with blue background are the residues extending outward in monomer; It is the LxxxA motif in the square frame.


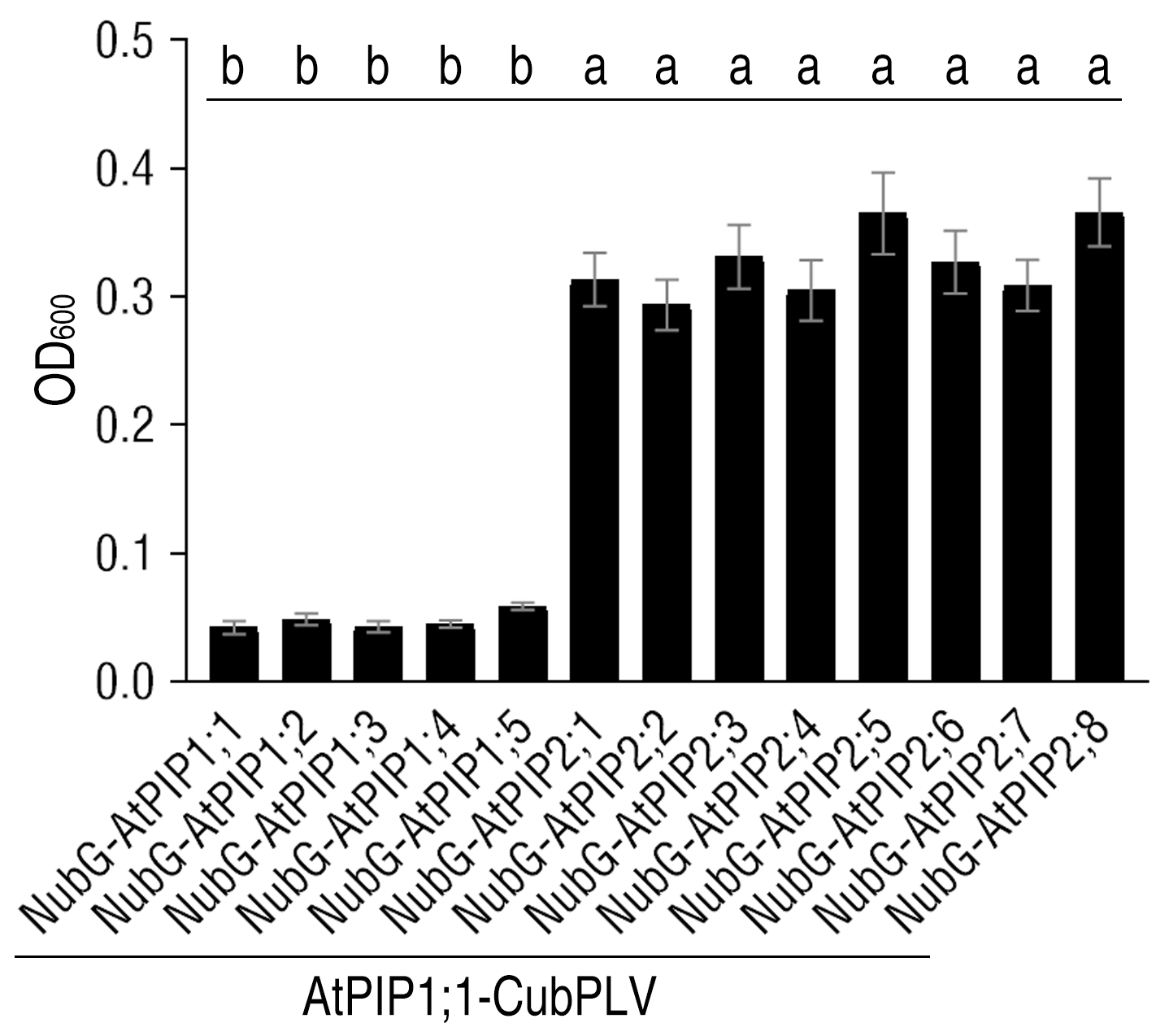


**Supplemental Figure 4:** The yeast concentrations of SUB Y2H positive transformants of AtPIP1;1-CubPLV and NubG-AtPIPs after a 48 hours culture in liquid SD-WLAH medium. Data shown are the means ±SDs. Different letters indicate significant differences in multiple comparisons of each pf values using an one-way ANOVA analysis, P<0.01. For each sample, triplicates (technical replicates) were measured and the experiments were repeated three times.


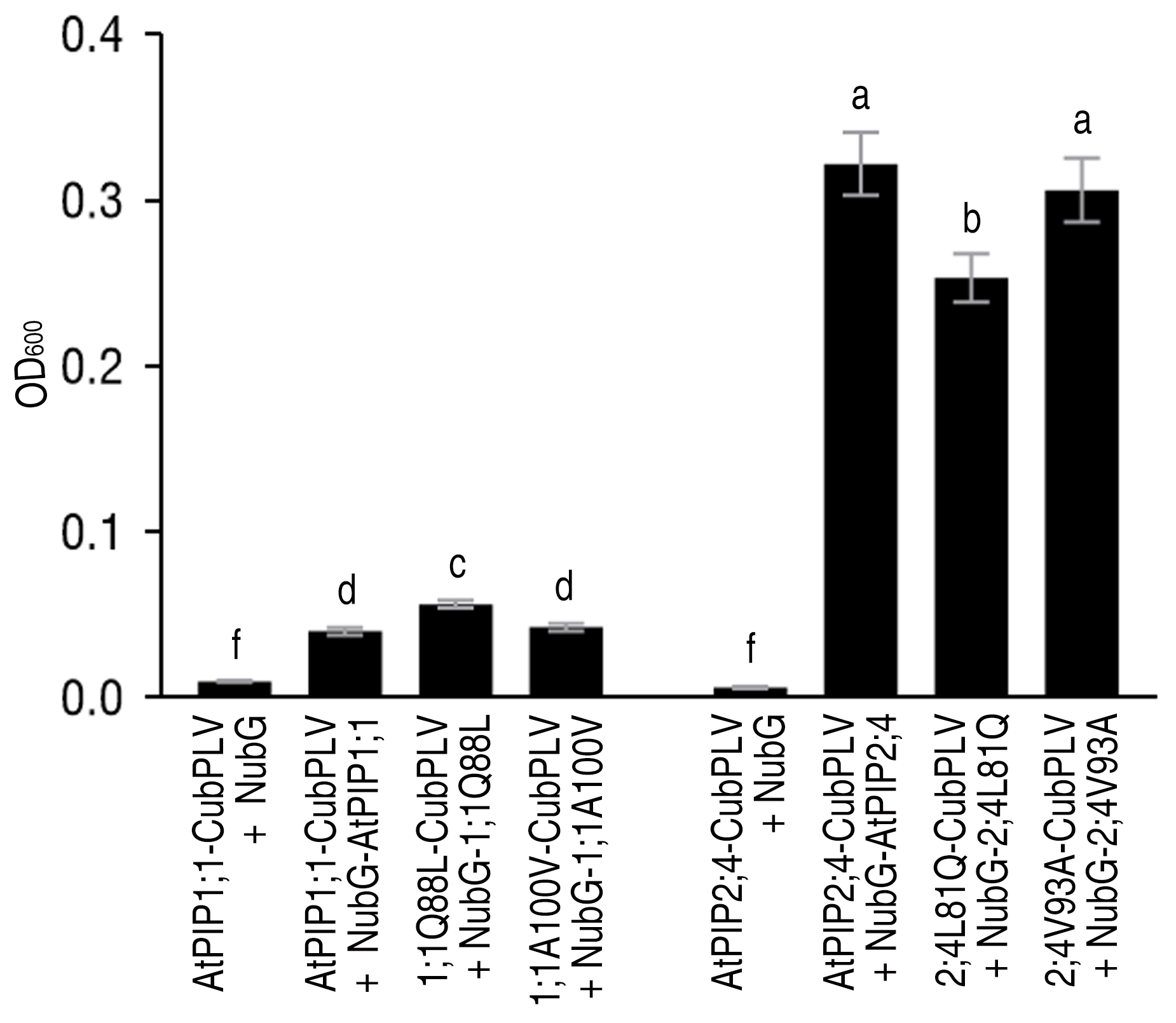


**Supplemental Figure 5:** The yeast concentrations of SUB Y2H positive transformants of AtPIP1;1 mutants or AtPIP2;4 mutants after a 48 hours culture in liquid SD-WLAH medium. Data shown are the means ±SDs. Different letters indicate significant differences in multiple comparisons of each pf values using an one-way ANOVA analysis, P<0.05. For each sample, triplicates (technical replicates) were measured and the experiments were repeated three times.


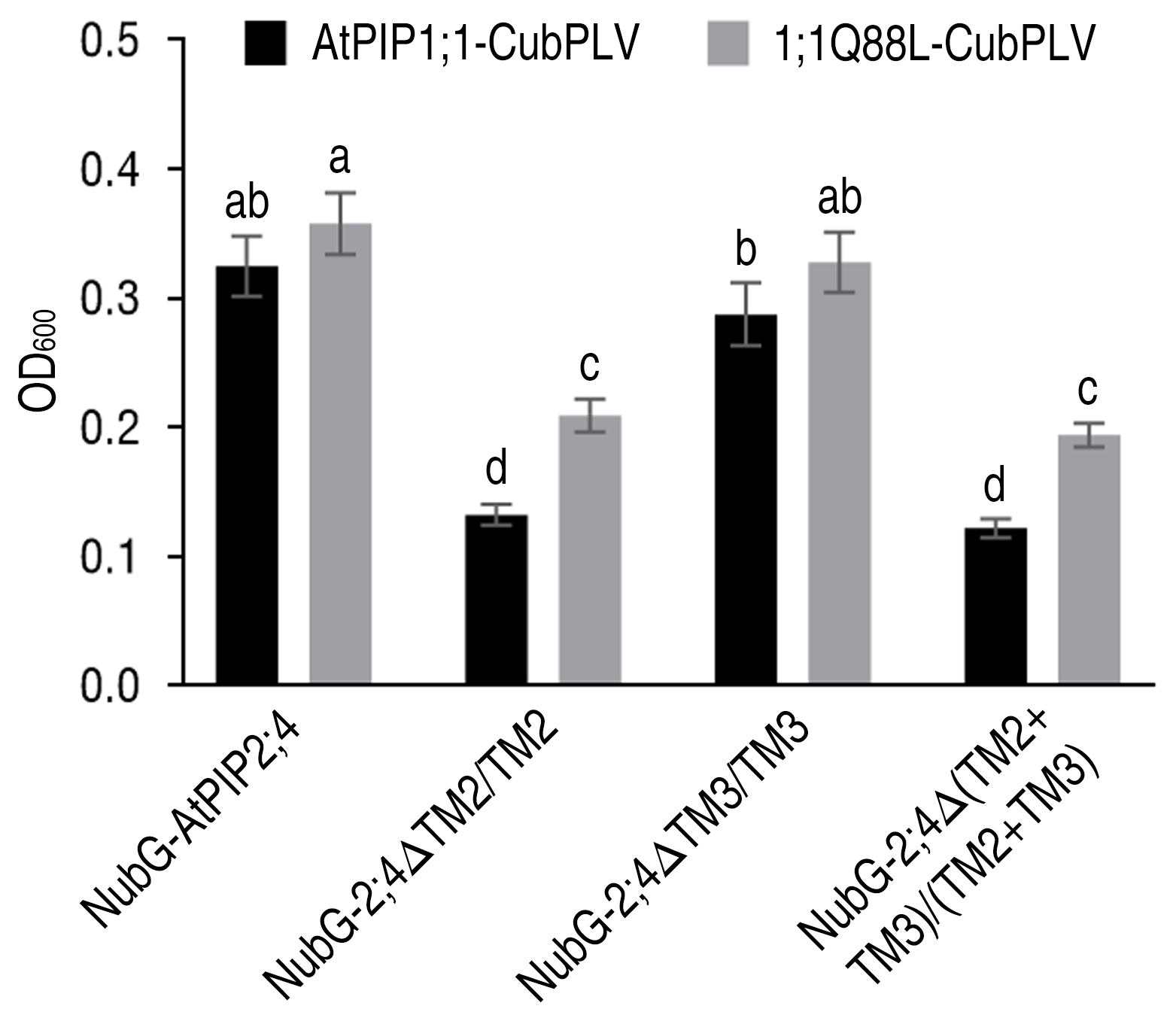


**Supplemental Figure 6:** The yeast concentrations of SUB Y2H positive transformants of AtPIP1;1-CubPLV/1;1Q88L-CubPLV and NubG-AtPIP2;4 or AtPIP2;4 mutants after a 48 hours culture in liquid SD-WLAH medium. Data shown are the means ±SDs. Different letters indicate significant differences in multiple comparisons of each pf values using an one-way ANOVA analysis, P<0.05. For each sample, triplicates (technical replicates) were measured and the experiments were repeated three times.


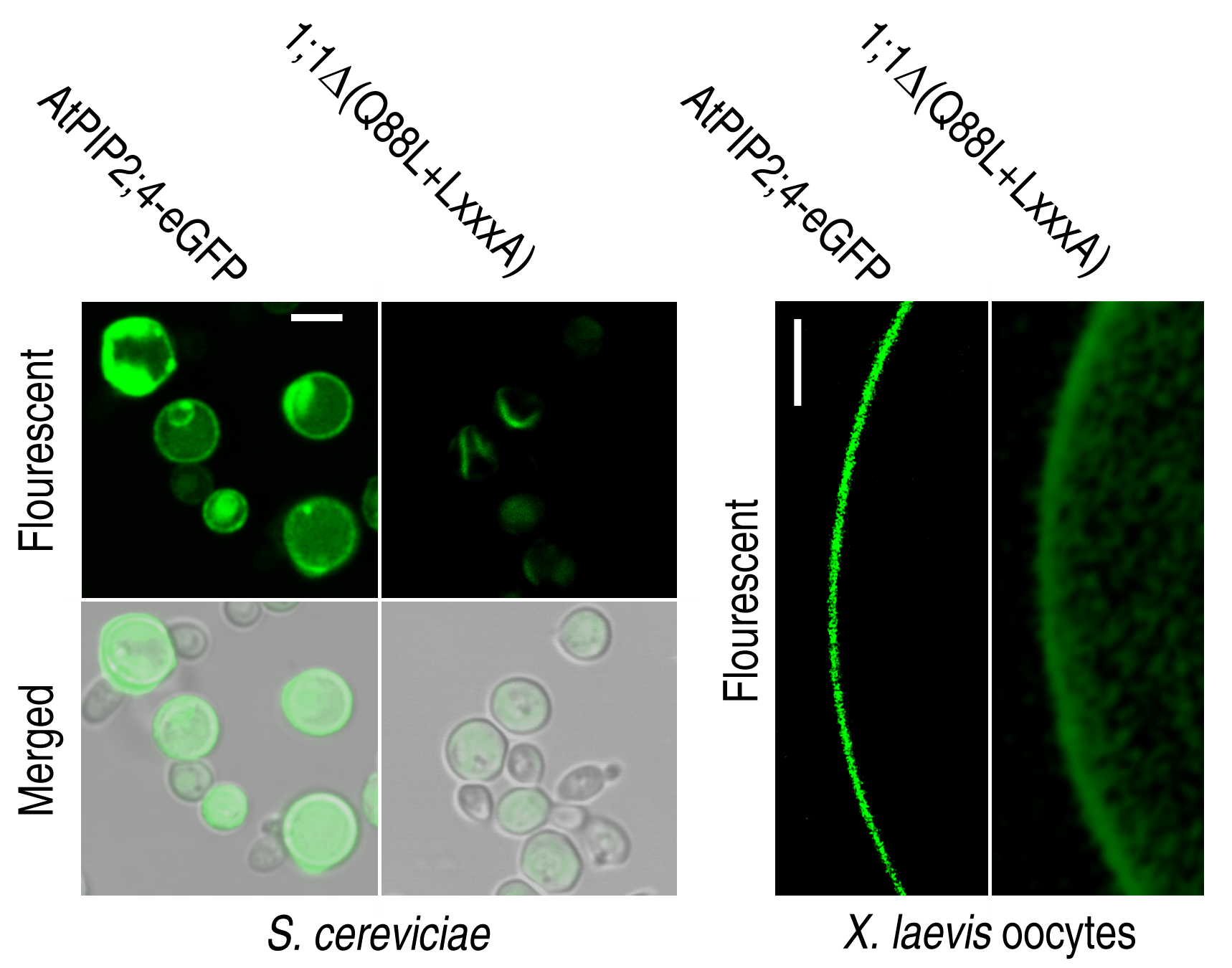


**Supplemental Figure 7:** Localization of 1;1Δ(Q88L+LxxxA) in *S. cereviciae* (left) and *X. laevis* oocytes (right). White scale bars represent the length of 5 μm (left) and 100 μm (right).
